# Study of the Cluster Thinning Grape as a Source of Phenolic Compounds and Evaluation of Its Antioxidant Potential

**DOI:** 10.3390/biom11020227

**Published:** 2021-02-05

**Authors:** Yolanda Carmona-Jiménez, Miguel Palma, Dominico A. Guillén-Sánchez, M. Valme García-Moreno

**Affiliations:** Departamento de Química Analítica, Facultad de Ciencias, Instituto Universitario de Investigación Vitivinícola y Agroalimentaria (IVAGRO), Universidad de Cádiz, Campus Universitario de Puerto Real, 11510 Puerto Real, Cádiz, Spain; yolanda.carmona@uca.es (Y.C.-J.); miguel.palma@uca.es (M.P.)

**Keywords:** thinned grape, by-products, winemaking wastes, polyphenols, antioxidant activity, ripening stage

## Abstract

Thinning is a common viticulture practice in warm climates, and it is applied to increase the quality of the harvest. Thinning clusters are usually discarded, and they are considered another oenological industry waste. To valorize this by-product, the phenolic content and antioxidant activity of three red varieties (Tempranillo, Cabernet Sauvignon, and Syrah), thinned at three different times between veraison and harvest, were studied: the first at the beginning of the veraison stage, in a low ripening stage; the second in an intermediate ripening stage; and, finally, the third sampling in the highest ripening stage. These by-products showed high values of total phenolic contents (10.66–11.75 mg gallic acid equivalent/g), which is of the same order as or even higher than that found in grape pomace. In thinned grape were identified 24 phenolic compounds, being the flavan-3-ols (catechin and epicatechin) of particular interest, with mean contents ranging from 105.1 to 516.4 mg/kg of thinned grape. Antioxidant activity similar to that of the vintage grape was found. It is concluded that thinned grape is a good source of phenolic compounds. Its content does not depend mainly on the grape variety; however, it has been possible to establish differences based on the maturity stage of the thinning grapes: the intermediate ripeness stage, with a Brix degree in the range of 15–16 for this area, would be the optimum collection time for cluster thinning. In this intermediate ripeness stage, thinning grapes present a higher antioxidant activity and there is also appreciable anthocyanin content, which is not found for the lowest ripeness stage, since these samples present an intermediate composition in all the families of determined phenolic compounds: anthocyanins, flavonols, flavan-3-ols, cinnamic acids, and benzoic acids. It is important to note that the experiments in this study have been carried out with whole tinned grapes, without separating the skin or the seeds.

## 1. Introduction

Grapes are one of the main fruit crops in the world. In 2018, about 57% of the world’s grape production was used in winemaking [1]. The winemaking industry produces large quantities of waste materials, and, at present, only minimal amounts of this waste are up-graded or recycled [2]. Nevertheless, in recent years, interest has been increasing in the possible use of vinification by-products for the production of high-value-added natural compounds [3,4,5,6].

By-products such as grape pomace have received significant attention, and numerous studies have been carried out on the use of grape pomace as a source of phenolic compounds, which can be used as food supplements, as new functional foods, or in the production of phytochemicals [7,8,9,10,11,12,13,14,15,16,17,18,19,20,21]. Phenolic compounds show antioxidant activity, and this is associated with beneficial effects on human health [22,23,24,25].

However, there are other by-products from the wine industry that have not received much attention; this is the case of the grapes obtained by cluster thinning. Thinning is a common viticulture practice applied to regulate the yield levels with the aim of increasing the quality of the harvested grape and reducing overcropping in areas where regulation imposes a limit on production yields. Cluster thinning consists of removing a proportion of the developing grape clusters (usually between 30 and 40%) to achieve a better-balanced vine. Factors such as crop level and the ratio between leaf area and total fruit weight per plant are regarded as being essential to control and ensure the correct maturity and development of the berries [26]. Cluster thinning influences the yield reduction, advances grape maturity, improves the phenolic content of the grape, and influences the volatile profile of wine. In addition, this technique tends to diminish acidity and increase soluble solids [27,28,29,30,31]. Therefore, despite the economic impact of thinning, this process is a very common activity in some warm-climate producer areas. 

The treatment time of cluster thinning has a significant impact on berry growth and grape composition [32]. Removal of the crop early in the season may not lead to the desired result because the reduced sink size might in turn lead to lower leaf photosynthesis rates, which may result in the remaining berries not having extra sugar available for import. If, however, photosynthesis remains unchanged, surplus photo-assimilates could also be used to fuel more shoot (and root) growth. This growth would counteract the benefits of a lower crop load because of its negative effect on vigor [33]. Many authors agree that the most effective time to carry out cluster thinning is the veraison, since at this time, the vegetative stop is reached and the tips of the vine shoot are not active and, therefore, the sugars synthesized by the leaves are accumulated only in clusters [34].

Thinned clusters are usually discarded and left on the ground in the vineyard [35]. To date, very little research has been directed toward the use or study of thinned grape. We find studies of the evolution of the phenolic content during the ripening of the grape, focused mainly on the study of the skin and seeds of the grape but not on the thinning grape itself [31,32,33,36,37,38,39,40,41]. Conversion of the cluster thinning by-products into a new material resource would lead to improved efficiency in the use of natural resources and revalorization of the thinned grape. It is necessary to characterize thinned grapes to exploit this resource in the best possible way. 

The aim of this study was to evaluate the potential use of cluster thinning as a source of bioactive compounds, specifically of phenolic and antioxidant compounds. 

## 2. Materials and Methods

### 2.1. Chemicals

Standards of phenolic compounds were purchased from Fluka (Buchs, Switzerland), Sigma-Aldrich (St. Louis, Mo, USA), and Merck (Darmstadt, Germany). 1,1-Diphenyl-2-picrylhydrazyl (DPPH) was provided by Sigma-Aldrich (St. Louis, Mo, USA). All other chemicals and solvents used for UPLC were purchased from Panreac (Barcelona, Spain). Ultrapure water was obtained from a Milli-Q system supplied by Millipore (Bedford, MA, USA).

### 2.2. Samples

This study was carried out on cluster thinning waste collected from a vineyard located in the Jerez area in the southwest of Spain. To limit the influence of external factors and to allow a better comparison between results, all samples shared the same geographical area and viticultural practices. In this vineyard, the crops are only irrigated according to water stress. Three of the most cultivated red grape varieties in this area were evaluated: Tempranillo, Cabernet Sauvignon, and Syrah. Sampling of manual cluster thinning was carried out at three different times between veraison and harvest: at the beginning of the veraison stage, in a low ripening stage (LRS); one in an intermediate ripening stage (IRS); and finally one sampling in the highest ripening stage (HRS) before the harvest moment. Samples of thinned clusters of each variety were taken in situ randomly from all over the growing area of the vineyard in an effort to obtain representative samples.

### 2.3. Sample Preparation

A total of 5 kg of each sample was transferred to the laboratory. The clusters were destemmed and grapes mixed manually. A proportion of each sample was pressed, and several control parameters were measured on the must. The remaining proportion of the berries was triturated with a conventional beater until a homogeneous sample was obtained for analysis. 

Extraction of the phenolics compounds was carried out on 1 g samples according to the method reported by Carrera et al. (2012) [42], with one modification: an ethanol/water mixture (50:50) was used as the solvent without acidification. A UP200S sonifier (200 W, 24 kHz; Hielscher Ultrasonics, Teltow, Germany) was used with 100% amplitude and a duty cycle of 1.0 s; this was immersed in a water bath coupled to a temperature controller at 10 °C (Frigiterm, J.P. Selecta, Barcelona, Spain). All extractions were carried out in duplicate. The extracts were frozen and stored at −20 °C prior to analysis. 

### 2.4. Control Parameters

pH, total soluble solids, and titratable acidity of the musts of cluster thinning grapes were measured as control parameters to determine the state of the grapes. The total soluble solids were obtained using an automatic density meter, model DMA 4500 M from Anton Paar (GmbH, Graz, Austria), and the values were expressed as Brix degree (°Bx). Titratable acidity was estimated according to the official method recommended by the International Organization of Vine and Wine (OIV), method OIV-MA-AS313-01. An automatic analyzer for the determination of pH and acidity in wine and must (model pH-Matic 23 from Crison Instruments S.A., Arella, Barcelona, Spain) was used, and the results of titratable acidity were expressed as g/L of tartaric acid. 

### 2.5. Total Phenolic Content

Firstly, general measurements of the total phenolic content were carried out. The total anthocyanin content (TAC), the total tannin content (TTC), and the total phenolic content (TPC) were determined spectrophotometrically. The TAC was measured at 520 nm and pH = 1, and the results are expressed as mg of malvidin equivalents (ME) per kg of grapes. Quantification of the TTC was carried out by the precipitation of condensed tannins with methyl cellulose [43,44], and the results were expressed as mg of catechin equivalents (CE) per g of thinned grape. The TPC was obtained by measuring the absorbance at 280 nm, and the results were expressed as mg of gallic acid equivalents (GAE) per g of thinned grape. All measurements were carried out on a V-530 UV-Vis spectrophotometer (Jasco, Madrid, Spain). Calibration curves were obtained using standard solutions of gallic acid, malvidin, and catechin, respectively.

### 2.6. Individual Phenolic Compounds

The characterization of individual phenolic compounds was achieved by ultraperformance liquid chromatography (UPLC). All samples were filtered through 0.22 µm membrane filters prior to analysis. Benzoic acids, cinnamic acids, and flavan-3-ols were determined with a binary phase of A (3% acetonitrile, 2% acetic acid, 95% water) and B (85% acetonitrile, 2% acetic acid, 13% water). Analyses were performed on a Waters Acquity UPLC system coupled to a photodiode array detector and a fluorescence detector. A Waters Acquity UPLC BEH C18 column, 100 mm length × 2.1 mm inner diameter, with 1.7 µm particle size, was used. The column temperature was maintained at 47 °C, the injection volume was 2.5 µL, and the flow rate was 0.7 mL/min. The gradient (6.5 min) was as follows: 0 min, 100% A; 3 min, 90% A; 4 min, 90% A; 6.5 min, 25% A. 

Each compound was identified by comparing the peak retention times with those previously obtained by the injection of standards. Quantification was achieved from the calibration curves of standards using fluorescence chromatograms at λ excitation/emission of 290/350 nm for benzoic acids and catechins and 340/420 nm for cinnamic acids. 

Flavonols and anthocyanins were determined on an Elite UPLC LaChrom Ultra system (VWR Hitachi, Tokyo, Japan). Prior to analysis, the samples were acidified with HCl to pH = 2 and incubated at room temperature for 3 h. The solvents used were acidified water with 5% formic acid (solvent A) and methanol (solvent B). A Halo™ C18 Hitachi LaChrom column (100 mm length × 3 mm inner diameter, with 2.7 µm particle size) was used. The column temperature was 50 °C, the injection volume was 15 µL, and the flow rate was 1 mL/min. The gradient employed was as follows: 0 min, 15% B; 1.50 min, 20% B; 3.30 min, 30% B; 4.80 min, 40% B; 5.40 min, 55% B; isocratic elution of 55% B until 7.00 min. Quantification was carried out from the UV-Vis spectra at 360 nm for flavonols and 520 nm for anthocyanins. Each compound was identified by comparing the peak retention times with those previously obtained by the injection of standards. Calibration curves of standards were produced for quantification.

### 2.7. Antioxidant Activity

The simplified DPPH assay for wine and wine by-products [45] was used to determine the antioxidant activity of thinned grapes, and the results were obtained according to simplified assay conditions. Measurements were made on three aliquots of each sample after 4 h at 515 nm in a Cary 50 Bio spectrophotometer (Varian, Australia). The results are expressed as EC_20_ (the amount of sample necessary to decrease the initial DPPH concentration by 20%).

### 2.8. Statistical Analysis

All analyses were made in duplicate. The results are presented as the mean value (MV) ± standard deviation (SD). Data were analyzed by one-way ANOVA and the Fisher’s Least Significant Difference (LSD) test to estimate the differences between values for the samples tested, where statistical significance was declared at *p* < 0.05. Previously, Levene’s test was employed to estimate the difference between the standard deviations of variables at the 95.0% confidence level. Pearson’s correlation was obtained between different parameters. Principal component analysis was carried out. The results were processed using the Statgraphics Centurion XVII software.

## 3. Results and Discussion 

### 3.1. Control Parameters

It can be seen from the results in Table 1 that the °Bx and acidity reveal different stages of maturity for the different samplings. The maturity stage is commonly measured by the values of total soluble solids (°Bx) and acidity. Higher total soluble solids and lower acidity values indicate more advanced maturation states. The changes in maturation are due to climatological factors and intrinsic characteristics inherent in each variety. Although samples were carried out close to the veraison stage, different levels of ripening were found in the three samplings. It is important to bear in mind that during the veraison stage the grapes are subject to significant changes in their composition in a very short period of time. Different stages of maturation close to the veraison stage provide a wide variety of sampling points and enable an evaluation of the potential of the thinning process in different ripeness stages. 

The LSD Fisher test performed on the data of the measured control parameters (Table 1) indicates that there are no statistically significant differences, with a confidence level of 95%, between the mean data obtained for the variables studied by varieties. However, different groups are identified, at a confidence level of 95%, if the variable studied is the ripening stage of the grapes. The test shows two groups for the pH variable and three groups for the °Bx and the total acidity.

### 3.2. Total Phenolic Content and Antioxidant Activity

#### 3.2.1. Total Phenolic Content

The global parameters, TAC, TTC, and TPC, related to the total phenolic content have been determined in the thinning grape samples for the variables studied. The average values obtained by variety and maturation stage are shown in Table 2.

In relation to the TAC, a wide range of levels was found, with amounts ranging from 0.02 to 1.10 mg of ME/g of thinned grape. The mean values obtained were 0.48 mg of ME/g of thinned grape for Tempranillo and Cabernet Sauvignon and 0.35 mg of ME/g of thinned grape for the Syrah variety. In literature, different results have been found in the case of grape pomace but they have been generally around this range. Ryan and Revilla [36] observed an increase in the content of total anthocyanins (expressed as mg malvidin 3-O-glucoside equivalent per kg of grapes) with the increase in sugar content during the ripening of the Tempranillo and Cabernet Sauvignon varieties. For Cabernet Sauvignon, the TAC values in our study varied between 264 and 847 mg/kg of grapes for the sugar values considered and between 254 and 732 mg/kg for the Tempranillo variety. Vian et al. [37] observed a similar behavior in the skins of the Syrah variety and De la Cerda-Carrasco et al. [8] in the dry pomace obtained from the Cabernet Sauvignon variety. 

It is not possible to establish differences between varieties, but there are statistical differences with respect to ripening stage. Significant changes were found between all the different stages. It is interesting to note that for the LRS samples (with a °Bx range of 10–12), anthocyanins were barely present, whereas for IRS samples (15–16 °Bx), the content became significant. This increase is part of the grape ripening process itself. During veraison, a loss of chlorophyll, an increase in the deformity of the berry, and the accumulation of sugar in the skin of the grapes, together with the formation of colored pigments, are observed [38]. 

The TTC study showed average amounts of 8.07–10.79 mg of CE/g of thinned grape. Once again, it was not possible to establish statistically significant differences between varieties. However, differences due to the maturation stage were evident. A marked decrease in tannin content was found between the LRS and HRS, in agreement with some authors [39] and in disagreement with others; there are other authors who have observed the different behavior: an increase in the total tannin content [40] or a slight change during the ripening period [41]. 

The study of the TPC revealed average contents of 11.02 mg of GAE/g of thinned grape for the Tempranillo variety and 11.75 and 10.66 mg of GAE/g of thinned grape for Cabernet Sauvignon and Syrah, respectively. These contents are within the range found by other authors in the study of grape pomace. For example, Ruberto et al. (2007) [2] found contents in the range 6.91–49.33 mg of GAE/g of dry grape pomace in different grape pomaces from Sicilian red grapes. De la Cerda-Carrasco et al. (2015) [8] found 15 mg of GAE/g of dry pomace for Cabernet Sauvignon cultivated in Chile. This means that the TPC of the thinned grape is comparable to the content found in grape pomace. Furthermore, when comparing the results for the thinned grape with those found in the literature for grape pomace, it is important to bear in mind that the grape has been thoroughly analyzed, including its aqueous content, so the concentration could be much greater if samples are dehydrated as pomace.

Results of an ANOVA test showed that, in general, it is not possible to identify differences between varieties in terms of their total phenol content. However, significant differences at the 95% confidence level were found in relation to the maturation stage. The samples corresponding to those with lower and higher maturation stages (LRS and HRS, respectively) contained the highest levels of TPC, and differences between them were not found. However, the samples with intermediate maturation (IRS) had the lowest TPC with statistical significance (Fisher’s LSD test). This trend may seem strange at first glance, but it can be explained by the phenolic changes that occur in the grape during maturation. It is known that during the maturation process, the tannin content decreases and the content of other phenolic compounds increases; this is the case for anthocyanins. Thus, intermediate maturation (IRS), which can be considered as having a °Bx range between 15 and 16, may be a point where the increase in anthocyanins still does not compensate for the decline in tannins.

#### 3.2.2. Antioxidant Activity

The antioxidant activity was studied by the simplified DPPH assay, and the results are shown in Table 2. The results are expressed as EC_20_ (i.e., amount of sample necessary to decrease the initial DPPH concentration by 20%) expressed as mg of thinned grape/mg of DPPH.

The mean EC_20_ values obtained were 4.04 for Cabernet Sauvignon, which has the highest antioxidant activity; 4.73 for Tempranillo; and 5.26 for Syrah, which has the lowest antioxidant activity. Although the thinned grapes of the Cabernet Sauvignon variety showed the highest antioxidant activity, it was not possible to establish statistically significant differences between the different varieties. 

In this case, significant differences in the maturation state were observed and samples with greater maturity had the lowest antioxidant activity. Fisher’s test shows significant differences between the sampling points. It identifies two groups: one group with greater antioxidant activity (lower EC_20_ values) where the samples taken in LRS and IRS are found, with lower IRS values than for LRS; and one group formed by the samples taken in HRS with lower antioxidant activity (higher values of EC_20_). These values are not consistent with the TPC values obtained, although they can be explained with the results obtained from the Pearson correlation test. 

The Pearson correlations (Table 3) showed a significant dependence of the antioxidant activity (EC_20_) on the TTC, with a negative correlation coefficient of −0.7616 (higher tannins contents show lower EC_20_ values, i.e., higher antioxidant activity) and a positive correlation coefficient with TAC (0.6452). However, a direct correlation between the TPC and EC_20_ was not found, as expected. Samples taken in IRS are those that present a more balanced composition, the mean values of TTC and TAC being therefore those that present higher values of antioxidant activity.

### 3.3. Individual Phenolic Compounds

In the study of the individual phenolic compounds, a total of 24 compounds were identified and quantified: three benzoic acids, a phenylethyl alcohol derivative, three cinnamic acids, two flavan-3-ols, five flavonols, and 10 anthocyanins (Table 4). 

The benzoic acids identified included protocatechuic acid, vanillic acid, and syringic acid. The total amount of benzoic acids (sum of individual benzoic acids) ranged from 60.5 to 827.4 mg/kg of thinned grape. Within this family, protocatechuic acid was the major compound found. It was not possible to quantify syringic acid in many of the samples. The cinnamic acids identified were chlorogenic acid, caffeic acid, and ferulic acid. The total cinnamic content (sum of individual cinnamic acids) ranged from 16.5 to 102.5 mg/kg of thinned grape. Caffeic acid was the major cinnamic acid in most of the samples, and chlorogenic acid was not quantified in any of the more ripened samples. Catechin and epicatechin were the flavan-3-ols identified. 

Large quantities of flavan-3-ols were found in all samples with mean contents ranging from 105.1 to 516.4 mg/kg of thinned grape. For most samples, around 60% of these quantities corresponded to catechin and 40% to epicatechin. These amounts are particularly relevant since according to literature references, there is additional interest in flavan-3-ol monomers due to their wide range of beneficial effects for human health [46].

The results of the statistical study showed that for the three families of compounds, namely benzoic acids, cinnamic acids, and flavonols, differences could not be established by grape variety. However, there are differences in the content according to the ripening stage. The samples in the lower ripening stage had significantly higher contents, whereas differences could not be established between intermediate- and high-ripeness samples.

Regarding flavan-3-ol monomers identified, their concentration declines drastically with the ripening of the grape. This behavior was also observed by Kyraleou et al. [39] in Syrah grapes grown in Greece, but not by Fanzone et al. in Malbec grape skins and seeds from Mendoza during ripening [33]. This decrease in the content of flavan-3-ols is due to the programmed oxidation of these compounds in the natural development of the seed toward a natural loss of astringency [47].

Five main flavonols were found in the thinned grapes studied: myricetin-3-glucuronide, myricetin-3-glucoside, quercetin-3-glucuronide, querecetin-3-rutinoside, and kaempherol-3-glucoside. The total flavonols (sum of individual flavonols) ranged from 48.5 to 85.0 mg/kg of thinned grape. Similar proportions were found for the different flavonols quantified, but in some samples, the contents of myricetin and kaempherol derivatives are worth highlighting. Significant differences for flavonols were not found by variety. The HRS showed the highest flavonol contents. Significant differences were not found in the contents for the IRS and LRS. Significant correlation coefficients were obtained between myricetin-3-glucuronide, kaempherol, and °Bx values (0.86, 0.86, and 0.87), whereas significant correlations were not found between the other flavonols. The concentration values obtained are significantly lower than those obtained by other authors for berries skins during ripening [31].

A total of 10 anthocyanins were identified, five of which were glycosylated anthocyanins and the other five were derivatives of these compounds. The sum of the quantified anthocyanins (total anthocyanins) gives an average content in the range of 71.2–1064.9 mg/kg of thinned grape. These results are consistent with those found on studying the TAC by spectrophotometry. Malvidin derivatives are the main anthocyanins present, with the contents of malividin-3-glucoside and malvidin-3-coumaroyl glucoside especially noteworthy. Once again, differences could not be established by variety for this family of compounds. 

In terms of maturation, however, significant differences were found between the different states considered. The concentration of all determined anthocyanins increases with the state of maturation, with malvidin-3-glucoside and malvidin-3-trans-p-coumaroylglucoside being the most abundant. Other authors have also observed the same behavior in the skins and seeds of different grape varieties [36,37,48]. The synthesis of anthocyanins in grapes during ripening depends on many factors: viticulture practice (cultivar, fertilizer, water regimes), environmental factors, the own genetic regulation of the variety, or the presence of precursor compounds such as carbohydrates. Carbohydrates are precursor compounds of anthocyanins. Therefore, high concentrations of carbohydrates favor the formation of anthocyanins [49]. 

The values found by us are lower than those found by other authors for the same varieties during the ripening stage. For example, Vian et al. [37] found maximum values of malvidin-3-glucoside in Syrah grapes, around 750 mg/kg of grapes, along with 250 mg/kg of delphinidin-3-glucoside, 220 mg/kg of petunidin-3-glucoside, and 200 of peonidin-3-glucoside, while the maximum values found by us for Syrah grapes are 188 mg/kg, 21 mg/kg, 25 mg/kg, and 78 mg/kg of thinned grape, respectively. The reason for this difference in values is the fact that we are evaluating the anthocyanin content in the whole grape and not in the skin or seeds of the grapes. 

**Figure 1:**
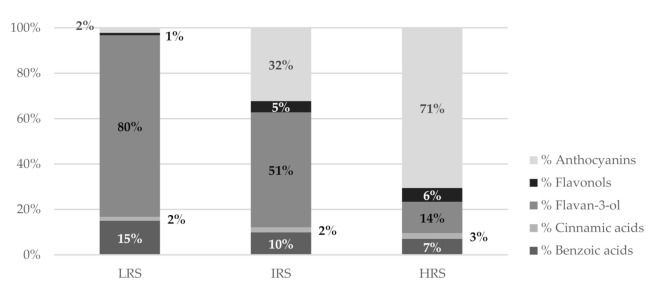
Percentages of the different families of phenolic compounds depending on their ripening stage.

The study of individual phenolic compounds, in agreement with the results obtained in the study of the general parameters, revealed on the one hand the presence of significant amounts of phenolic compounds, with their contents in monomers of catechin and epicatechin being of particular interest, and on the other hand that the content did not depend to a great extent on the selected grape variety but on its exact ripening stage. The evolution of the different phenolic families in relation to the ripening stage of the cluster thinning grapes is represented in Figure 1. 

**Figure 2:**
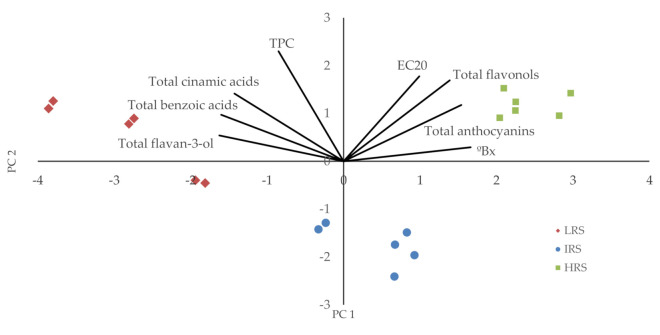
Principal component analysis.

The results of a principal component analysis (PCA; Figure 2) showed that the TPC, total benzoic acids, total cinnamic acids, and total flavan-3-ols are related and contribute negatively to component 1, whereas EC_20_, °Bx, TFC, and TAC contribute positively to component 1. All parameters contribute positively to component 2. This analysis reveals how the samples are grouped according to their °Bx range. For the more immature samples, the benzoic, cinnamic, and flavan-3-ols acids have more weight. In the case of more mature samples, the values of flavonols, anthocyanins, and EC_20_ (lower antioxidant activity) contribute more. In samples of intermediate maturity, all of the parameters are relevant. The results show that the intermediate ripeness stage, with a °Bx in the range of 15–16 for this area, would be the optimum collection time for cluster thinning. In the intermediate ripeness stage, a higher antioxidant activity was found than in the highest ripeness stage considered and there is also appreciable anthocyanin content, which is not found in the lowest ripeness stage.

## 4. Conclusions

In conclusion, it has been demonstrated that the thinned grapes have a significant content of phenolic compounds. The thinned grapes could therefore be used as a source of this type of compound. It is not necessary to extract the seeds or separate the skin. Thinning grapes are by themselves an interesting by-product with high added value thanks to the diversity and concentration of phenolic compounds present in them.

The phenolic content depends mainly on the exact ripening stage when thinning is carried out and not on the grape variety. Small differences in ripeness lead to significant differences in the phenolic content. To ensure a higher phenolic content that is rich in terms of variability and the maximum antioxidant activity, it is necessary that the grapes have an intermediate ripening stage. Thinned clusters must be harvested only several days after the veraison stage to ensure a richer variety of phenolic compounds and the maximum antioxidant activity.

## Figures and Tables

**Table 1:** Control parameters presented as MV ± SD. Total acidity is expressed as g/L of tartaric acid.

Variety	pH	Brix Degree	Total Acidity
*Tempranillo*			
LRS	2.81 ± 0.02 ^a^	12.11 ± 0.00 ^a^	24.59 ± 0.20 ^c^
IRS	3.17 ± 0.06 ^b^	15.76 ± 0.01 ^b^	7.78 ± 0.45 ^b^
HRS	3.03 ± 0.01 ^b^	19.09 ± 0.01 ^c^	6.80 ± 1.23 ^a^
*Cabernet Sauvignon*			
LRS	2.71 ± 0.02 ^a^	9.75 ± 0.00 ^a^	32.09 ± 0.54 ^c^
IRS	3.02 ± 0.03 ^b^	16.06 ± 0.00 ^b^	15.46 ± 0.41 ^b^
HRS	3.10 ± 0.03 ^b^	20.04 ± 0.00 ^c^	9.46 ± 0.37 ^a^
*Syrah*			
LRS	2.73 ± 0.01 ^a^	10.11 ± 0.00 ^a^	31.39 ± 0.46 ^c^
IRS	3.05 ± 0.04 ^b^	15.38 ± 0.01 ^b^	15.22 ± 0.96 ^b^
HRS	3.06 ± 0.03 ^b^	17.97 ± 0.00 ^c^	9.60 ± 1.72 ^a^

Values followed by the same letter (a, b or c) within a column are not significantly different at *p* ≤ 0.05 (Fisher’s LSD). LRS: low ripening stage; IRS: intermediate ripening stage; HRS: high ripening stage.

**Table 2:** General phenolic index studied for cluster thinning grapes expressed as MV ± SD: TAC expressed as mg of ME/kg of thinned grape, TTC expressed as mg of CE/g of thinned grape, TPC as mg of GAE/g of thinned grape, and EC_20_ as mg of thinned grape/mg of DPPH.

Variety	TAC	TTC	TPC	EC_20_
*Tempranillo*				
LRS	130 ± 18 ^a^	12.52 ± 0.24 ^c^	11.94 ± 0.16 ^b^	4.37 ± 0.04 ^a^
IRS	312 ± 8 ^b^	8.32 ± 0.44 ^b^	9.69 ± 0.37 ^a^	4.09 ± 0.04 ^a^
HRS	995 ± 60 ^c^	7.63 ± 0.26 ^a^	11.44 ± 0.26 ^b^	5.72 ± 0.21 ^b^
*Cabernet Sauvignon*				
LRS	41 ± 9 ^a^	14.00 ± 0.19 ^c^	12.88 ± 0.31 ^b^	3.61 ± 0.05 ^a^
IRS	293 ± 23 ^b^	12.03 ± 0.31 ^b^	11.25 ± 0.14 ^a^	2.47 ± 0.15 ^a^
HRS	1100 ± 61 ^c^	6.34 ± 0.38 ^a^	11.11 ± 0.38 ^b^	6.02 ± 0.13 ^b^
*Syrah*				
LRS	17 ± 4 ^a^	9.10 ± 0.15 ^c^	10.92 ± 0.12 ^b^	5.00 ± 0.02 ^a^
IRS	349 ± 21 ^b^	8.66 ± 0.36 ^b^	9.65 ± 0.51 ^a^	3.94 ± 0.05 ^a^
HRS	675 ± 11 ^c^	6.45 ± 0.12 ^a^	11.41 ± 0.34 ^b^	6.85 ± 0.39 ^b^

Values followed by the same letter (a, b, or c) within a column are not significantly different at *p* ≤ 0.05 (Fisher’s LSD). TAC: total anthocyanin content; TTC: total tannin content; TPC: total phenolic content; EC_20_: efficient concentration at 20%. LRS: low ripening stage; IRS: intermediate ripening stage; HRS: high ripening stage.

**Table 3:** Pearson correlations coefficients and *p*-values (in parentheses) at the 95% confidence level.

	TAC	TTC	TPC	EC_20_
TAC		−0.7373 (0.0005)	−0.0960 (0.7046)	0.6452 (0.0038)
TTC	−0.7373 (0.0005)		0.5469 (0.0188)	−0.7616 (0.0002)
TPC	−0.0960 (0.7046)	0.5469 (0.0188)		0.0335 (0.8949)
EC_20_	0.6452 (0.0038)	−0.7616 (0.0002)	0.0335 (0.8949)	

*p*-values below 0.05 indicate statistically significant non-zero correlations at the 95.0% confidence level. TAC: total anthocyanin content; TTC: total tannin content; TPC: total phenolic content; EC_20_: efficient concentration at 20%.

**Table 4:** Phenolic compound contents in thinning grapes, expressed as the mean ± SD (mg/kg of thinning grapes).

Phenolic Compounds	TM LRS	TM IRS	TM HRS	CS LRS	CS IRS	CS HRS	SY LRS	SY IRS	SY HRS
Benzoic acids, cinnamic acids, and flavan-3-ols									
Protocatechuic acid	296.89 ± 16.08 ^b^	39.55 ± 0.75 ^a^	58.65 ± 1.85 ^a^	325.67 ± 4.51 ^b^	70.84 ± 24.16 ^a^	107.78 ± 7.38 ^a^	151.42 ± 5.69 ^b^	34.97 ± 0.78 ^a^	105.56 ± 6.72 ^a^
Tyrosol	513.56 ± 29.62 ^b^	44.22 ± 2.71 ^a^	N.Q.	468.04 ± 40.79 ^b^	85.79 ± 6.96 ^a^	N.Q.	307.14 ± 8.22 ^b^	51.38 ± 0.55 ^a^	N.Q.
Vanillic acid	16.98 ± 0.06 ^b^	5.33 ± 0.40 ^a^	N.Q.	22.06 ± 0.14 ^b^	10.96 ± 0.08 ^a^	N.Q.	15.54 ± 0.17 ^b^	6.49 ± 0.13 ^a^	N.Q.
Syringic acid	N.Q.	N.Q.	1.84 ± 0.11	N.Q.	N.Q.	N.Q.	N.Q.	N.Q.	N.Q.
Chlorogenic acid	14.11 ± 0.53 ^a^	14.03 ± 4.74 ^a^	N.Q.	11.22 ± 1.56 ^a^	11.12 ± 1.78 ^a^	N.D.	N.Q.	10.63 ± 1.68 ^a^	N.Q.
Caffeic acid	81.45 ± 4.74 ^b^	6.44 ± 0.64 ^a^	21.09 ± 0.90 ^a^	76.94 ± 0.44 ^b^	12.23 ± 0.17 ^a^	21.04 ± 2.29 ^a^	42.30 ± 0.70 ^b^	N.Q.	12.36 ± 1.33 ^a^
Ferulic acid	6.90 ± 0.10 ^b^	6.25 ± 0.24 ^a^	21.16 ± 0.75 ^c^	7.56 ± 0.06 ^b^	6.36 ± 0.50 ^a^	N.D.	6.55 ± 0.10 ^b^	5.85 ± 0.70 ^a^	21.19 ± 0.90 ^c^
Catechin	1689.54 ± 116.41 ^c^	111.12 ± 1.20 ^b^	65.86 ± 3.24 ^a^	2881.42 ± 163.58 ^c^	1026.81 ± 76.80 ^b^	168.98 ± 2.05 ^a^	1493.32 ± 85.92 ^c^	357.35 ± 10.53 ^b^	94.63 ± 6.51 ^a^
Epicatechin	1091.25 ± 10.91 ^b^	64.25 ± 4.58 ^a^	39.21 ± 2.22 ^a^	2279.02 ± 5.58 ^b^	380.51 ± 30.14 ^a^	88.31 ± 1.26 ^a^	2297.10 ± 42.49 ^b^	281.97 ± 16.28 ^a^	90.44 ± 1.40 ^a^
Flavonols									
Myricetin-3-glucuronide	9.65 ± 0.12 ^a^	12.53 ± 0.50 ^b^	20.13 ± 1.35 ^c^	8.85 ± 0.25 ^a^	11.27 ± 0.23 ^b^	28.34 ± 0.77 ^c^	8.70 ± 0.30 ^a^	11.98 ± 1.43 ^b^	22.53 ± 0.89 ^c^
Myricetin-3-glucoside	13.62 ± 0.76 ^a^	15.12 ± 1.16 ^a^	16.03 ± 1.22 ^b^	17.05 ± 0.35 ^a^	15.09 ± 0.13 ^a^	21.36 ± 2.21 ^b^	15.67 ± 1.53 ^a^	10.63 ± 1.24 ^a^	17.57 ± 0.47 ^b^
Quercetin-3-glucuronide	8.65 ± 0.12 ^b^	7.42 ± 0.21 ^a^	8.47 ± 0.05 ^b^	7.99 ± 0.16 ^b^	6.97 ± 0.53 ^a^	8.50 ± 0.50 ^b^	8.18 ± 0.26 ^b^	6.90 ± 0.98 ^a^	8.17 ± 0.52 ^b^
Quercetin-3-rutinoside	8.64 ± 0.18 ^b^	8.18 ± 0.00 ^a^	9.67 ± 0.31 ^c^	8.75 ± 0.36 ^b^	7.51 ± 0.19 ^a^	9.38 ± 0.61 ^c^	8.63 ± 0.03 ^b^	7.68 ± 1.41 ^a^	9.08 ± 0.58 ^c^
Kaempherol-3-glucoside	7.95 ± 0.18 ^a^	13.00 ± 0.63 ^b^	21.76 ± 1.05 ^c^	8.25 ± 0.57 ^a^	10.08 ± 0.64 ^b^	17.43 ± 1.17 ^c^	8.07 ± 0.21 ^a^	12.41 ± 0.80 ^b^	19.51 ± 0.15 ^c^
Anthocyanins									
Delphinidin-3-glucoside	15.81 ± 0.07 ^a^	22.09 ± 0.78 ^a^	58.32 ± 4.97 ^b^	7.64 ± 0.68 ^a^	18.29 ± 0.28 ^a^	44.96 ± 2.79 ^b^	4.88 ± 0.16 ^a^	7.94 ± 0.12 ^a^	21.20 ± 0.62 ^b^
Cyanidin-3-glucoside	11.06 ± 0.26 ^a^	5.94 ± 0.18 ^a^	21.41 ± 0.47 ^b^	6.26 ± 0.00 ^a^	4.58 ± 0.04 ^a^	18.21 ± 0.19 ^b^	4.07 ± 0.05 ^a^	4.12 ± 0.19 ^a^	16.26 ± 0.39 ^b^
Petunidin-3-glucoside	15.65 ± 0.90 ^a^	23.78 ± 0.80 ^a^	64.67 ± 6.25 ^b^	6.89 ± 0.26 ^a^	15.90 ± 0.42 ^a^	38.51 ± 1.12 ^b^	5.93 ± 0.11 ^a^	13.45 ± 0.25 ^a^	24.79 ± 0.55 ^b^
Peonidin-3-glucoside	19.63 ± 0.34 ^a^	15.01 ± 0.67 ^a^	89.60 ± 2.87 ^b^	6.66 ± 0.25 ^a^	18.24 ± 0.45 ^a^	109.35 ± 1.22 ^b^	5.73 ± 0.10 ^a^	24.22 ± 1.73 ^a^	78.53 ± 0.21 ^b^
Malvidin-3-glucoside	41.34 ± 1.89 ^a^	97.19 ± 2.33 ^b^	328.23 ± 19.87 ^c^	19.13 ± 0.03 ^a^	93.21 ± 3.15 ^b^	335.20 ± 11.38 ^c^	17.74 ± 0.43 ^a^	100.40 ± 2.91 ^b^	171.40 ± 0.99 ^c^
Malvidin-3-acetylglucoside	9.69 ± 0.05 ^a^	34.48 ± 0.78 ^a^	69.50 ± 1.26 ^b^	16.60 ± 0.21 ^a^	109.61 ± 1.65 ^a^	265.41 ± 13.01 ^b^	8.39 ± 0.58 ^a^	60.98 ± 1.79 ^a^	99.30 ± 2.05 ^b^
Malvidin-3-cis-p-coumaroylglucoside	8.76 ± 0.12 ^a^	21.42 ± 1.14 ^b^	62.01 ± 4.02 ^c^	5.57 ± 0.13 ^a^	11.99 ± 1.10 ^b^	35.24 ± 0.74 ^c^	5.94 ± 0.21 ^a^	29.41 ± 1.70 ^b^	48.80 ± 1.60 ^c^
Malvidin-3-caffeoylglucoside	5.06 ± 0.24 ^a^	28.85 ± 1.25 ^b^	43.88 ± 5.49 ^c^	4.50 ± 0.05 ^a^	12.97 ± 1.24 ^b^	28.37 ± 1.44 ^c^	4.78 ± 0.13 ^a^	33.75 ± 1.01 ^b^	32.32 ± 3.34 ^c^
Petunidin-3-p-coumaroylglucoside	7.92 ± 0.54 ^a^	26.03 ± 1.45 ^b^	59.79 ± 0.85 ^c^	5.02 ± 0.20 ^a^	10.88 ± 2.31 ^b^	27.09 ± 2.27 ^c^	5.96 ± 0.42 ^a^	19.23 ± 4.16 ^b^	37.03 ± 0.66 ^c^
Malvidin-3-trans-p-coumaroylglucoside	10.62 ± 0.19 ^a^	70.78 ± 5.52 ^b^	224.78 ± 8.06 ^c^	6.52 ± 0.10 ^a^	30.90 ± 2.14 ^b^	162.55 ± 15.36 ^c^	7.77 ± 0.54 ^a^	58.58 ± 2.28 ^b^	187.70 ± 2.76 ^c^

N.D.: not detected; N.Q.: not quantified; mean values followed by the same letter (a, b, or c) within a row are not significantly different at *p* ≤ 0.05 (Fisher’s LSD); TM: Tempranillo; CS: Cabernet Sauvignon; SY: Syrah; LRS: low ripening stage; IRS: intermediate ripening stage; HRS: high ripening stage.

## Data Availability

Not applicable.
